# Host Genetics in Granuloma Formation: Human-Like Lung Pathology in Mice with Reciprocal Genetic Susceptibility to *M. tuberculosis* and *M. avium*

**DOI:** 10.1371/journal.pone.0010515

**Published:** 2010-05-06

**Authors:** Elena Kondratieva, Nadya Logunova, Konstantin Majorov, Mikhail Averbakh, Alexander Apt

**Affiliations:** Laboratory for Immunogenetics, Central Institute for Tuberculosis, Moscow, Russia; Institut de Pharmacologie et de Biologie Structurale, France

## Abstract

Development of lung granulomata is a hallmark of infections caused by virulent mycobacteria, reflecting both protective host response that restricts infection spreading and inflammatory pathology. The role of host genetics in granuloma formation is not well defined. Earlier we have shown that mice of the I/St strain are extremely susceptible to Mycobacterium tuberculosis but resistant to M. avium infection, whereas B6 mice show a reversed pattern of susceptibility. Here, by directly comparing: (i) characteristics of susceptibility to two infections in vivo; (ii) architecture of lung granulomata assessed by immune staining; and (iii) expression of genes encoding regulatory factors of neutrophil influx in the lung tissue, we demonstrate that genetic susceptibility of the host largely determines the pattern of lung pathology. Necrotizing granuloma surrounded by hypoxic zones, as well as a massive neutrophil influx, develop in the lungs of M. avium-infected B6 mice and in the lungs of M. tuberculosis-infected I/St mice, but not in the lungs of corresponding genetically resistant counterparts. The mirror-type lung tissue responses to two virulent mycobacteria indicate that the level of genetic susceptibility of the host to a given mycobacterial species largely determines characteristics of pathology, and directly demonstrate the importance of host genetics in pathogenesis.

## Introduction

A key feature of mycobacterial infections is formation of tissue granulomata whose anatomical locations and fine structure differ substantially depending upon the species of causative mycobacteria and the level of immune responses. Granuloma is considered as a battlefield between mycobacteria and host, providing both protective tissue reaction and inflammatory site where the pathology progresses [1], [2]. During *Mycobacterium tuberculosis* and *M. avium* infections in patients, granulomata are formed predominantly in the lungs, and eventually undergo necrosis and erode into bronchi, spreading mycobacteria and serving another biological function – horizontal transmission, which is beneficial for the parasite but deleterious for the host population [3]. Thus, understanding of granuloma formation, maturation, necrosis and, occasionally, healing is a hallmark of dissecting pathogenesis of mycobacterial diseases in general and tuberculosis (TB) in particular. This, in turn, is essential for developing new tools for TB control.

Much of what we know about TB immunity and genetics we have learned from experiments in inbred laboratory mice, which demonstrated that humans and mice are similar in the main features of the innate and adaptive immune responses to mycobacteria, that is, the protective role of CD4^+^ T cells, activated macrophages, IFN-γ, and TNF-α [4]. However, thus far modeling TB infection in mice created only limited amount of appreciable knowledge concerning pathogenesis of human TB. Moreover, mouse experimental models of TB were subjected to criticism as non-adequately mimicking the human disease. It was repeatedly put forward that there is no central necrosis in lung granulomata of TB-infected mice [5], [6] and that granulomatous zones remain aerobic in the lungs of mice, in contrast to humans [7], [8]. As discussed recently [9], these observations may be explained in the first instance by the choice of experimental mouse models. Mostly, corresponding data were obtained in mice of a TB-resistant mouse strain B6 or its derivatives, whereas mice of several genetically TB-susceptible strains repeat features of human TB pathology substantially more accurately [10], [11]. Nevertheless, an alternative way to study mycobacterial granuloma formation and development in mice was suggested and applied. In B6 and relative mouse strains *M. avium* causes lung granuloma with regular structure, sharing many features with human TB granuloma; thus, corresponding models of infection were used to study cellular and molecular interactions during mycobacterial granulomatosis [12], [13]. The authors accurately emphasized that care should be exercised when extrapolating their results to TB immunity, but did not touch the genetic aspect of the problem.

Recently we have shown that mice of the I/St strain, which are extremely susceptible to TB [14], are resistant to *M. avium* infection, whereas the opposite is true for the B6 strain [15]. In agreement with data obtained by Ehlers et al. [12], [13], general appearance of *M. avium*-induced lung granuloma in B6 mice closely resembled that of human TB lesions, but in I/St mice lung pathology was much milder and was not regularly shaped. On the other hand, *M. tuberculosis* caused a human-like pathology in I/St animals according to our observations [10]. Here, by directly comparing characteristics of susceptibility to two infections, architecture of lung granulomata assessed by immune staining, and expression of genes encoding regulatory factors of neutrophil response in the lung tissue, we demonstrate that genetic susceptibility of the host largely determines the pattern of lung pathology: mirror-type lung tissue responses develop in *M. avium-*susceptible B6 and *M. tuberculosis*-susceptible I/St mice following infection with the corresponding agent. Our results emphasize that reliability of infectious models critically depend upon genetics of the host.

## Results and Discussion

### Mirror phenotypes of susceptibility and resistance to *M. tuberculosis* and *M. avium* in I/St and B6 mice

**Figure 1:**
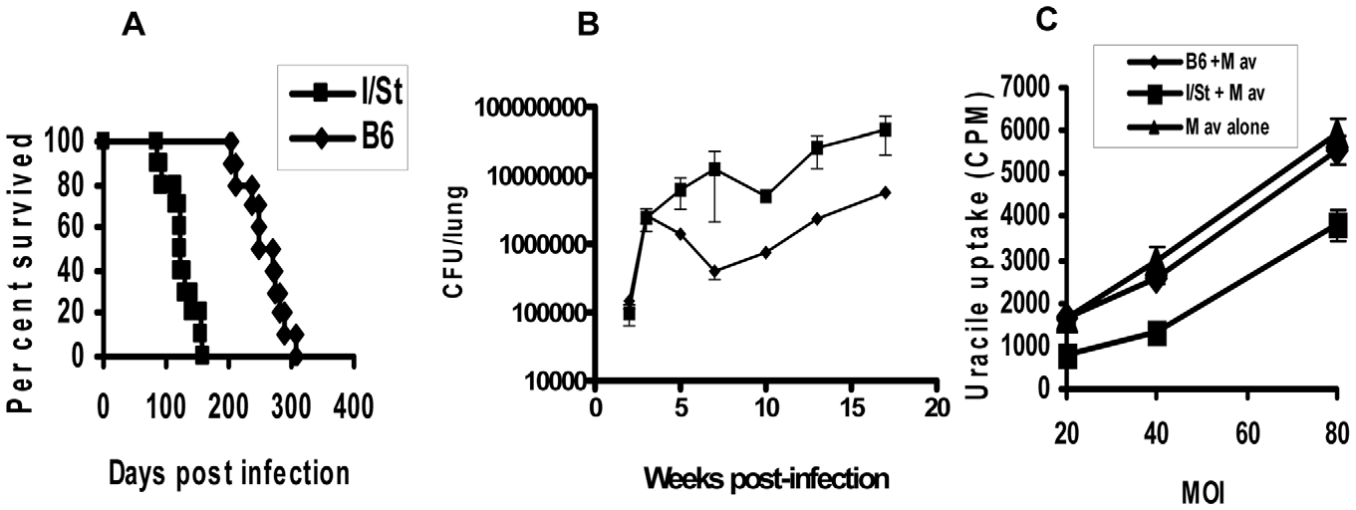
B6 mice are more resistant to *M. tuberculosis* infection compared to I/St mice. Their survival time (A, *P*<0.001, Gohen's criterion for survival curves) is longer and lung CFU counts (B, *P*<0.01-0.001 at different time points, ANOVA) are lower. Lung macrophages of I/St, but not of B6, mice inhibit multiplication of *M. avium* after in vitro infection within a high range of MOI (C). The rate of mycobacterial growth was measured by [^3^H]-uracil uptake at 72 h after establishing co-cultures. 1 µCi/well [^3^H]-uracil was added for the last 18 h of incubation. The wells containing mycobacteria alone at numbers corresponding to each MOI served as controls. Results obtained in one of three similar experiments are expressed as mean CPMs ± SD for triplicate cultures; interstrain differences are statistically significant (*P*<0.01, Mann-Whitney's U-test).

Previously we have demonstrated that I/St and B6 mice display, respectively, resistant and susceptible phenotype when infected with *M. avium* [15]. Using several infection routes and doses, we have shown also that I/St mice are susceptible to *M. tuberculosis* [14], [16], [17] and that their interstitial lung macrophages display an impaired capacity to inhibit *M. tuberculosis* growth [18]. Since not B6, but A/Sn, mice were always used in our previous TB studies as the resistant counterpart, in this work we directly compared disease progression in I/St and B6 mice after aerosol *M. tuberculosis* challenge. Groups of corresponding animals were infected with ∼10^2^ CFU of *M. tuberculosis* H37Rv, and lung CFU counts and survival of animals were assessed. I/St mice developed substantially more severe course of the disease, compared to B6 mice, both in terms of survival time (Fig. 1A, *P*<0.001, Gohen's criterion for survival curves) and CFU counts (Fig. 1B, *P*<0.01-0.001 at different time points, ANOVA).

Earlier we and others have shown that inability of B6 mice to control progression of *M. avium-*triggered disease largely depends upon the expression of a non-functional *Nramp1* ^s^ allele [15], [19]. A putative functional role of this gene as an endosome efflux pump that sequesters iron and, possibly, other divalent cations from the endosomal system in macrophages [20], [21] suggested that lung macrophages from B6 mice may less efficiently inhibit *M. avium* growth compared to I/St-*Nramp1^r^* macrophages. To evaluate this macrophage function, we applied the experimental system developed earlier for *M. tuberculosis* [18] to study *M. avium* multiplication following in vitro infection of lung macrophages from the two mouse strains. As shown in Fig. 1C, the ability to inhibit *M. avium* growth was almost lacking in B6 but readily expressed in I/St macrophages, opposite to their defective response against *M. tuberculosis* [18]. Thus, the genetically determined capacities to control TB and *M. avium*-triggered disease in these two mouse strains are indeed mirror-like.

### Architecture of lung pathology in susceptible and resistant mice: comparative aspects

**Figure 2:**
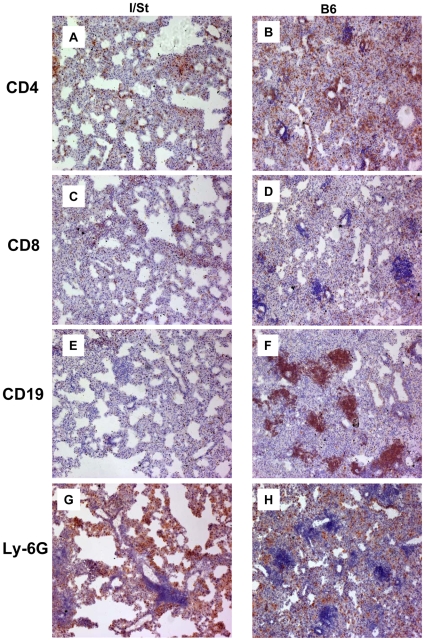
The picture of leukocyte infiltration of the lung tissue of I/St (left) and B6 (right) mice infected with 2×10^3^ CFU of *M. avium* via aerosol route 8 weeks earlier. Peroxidase immune staining with hematoxylin counter-staining (×150). Cell populations are indicated on the left side. See text for the description.

**Figure 3:**
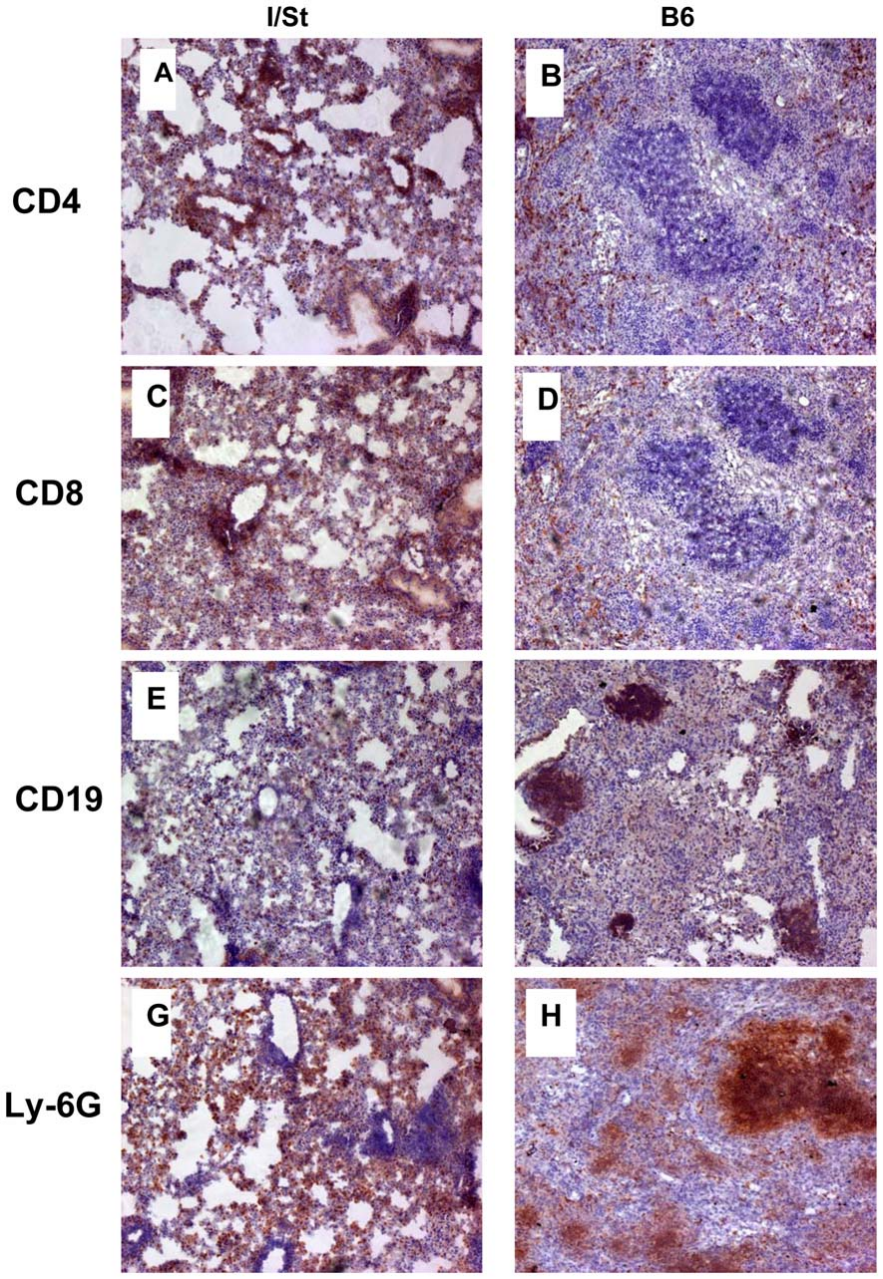
The picture of leukocyte infiltration of the lung tissue of I/St (left) and B6 (right) mice infected with 2×10^3^ CFU of *M. avium* via aerosol route 16 weeks earlier. See legend to Fig. 2.

In our recent studies we have characterized the general picture of lung pathology in *M. avium-*infected B6 and I/St mice [15], as well as the cellular composition of lung lesions in *M. tuberculosis*-infected I/St mice [10]. In order to complete our analysis of pathology caused by the two mycobacterial species in genetically susceptible and resistant hosts, we compared dynamic pictures of lung infiltration with lymphoid cells in *M. avium-*infected B6 and I/St mice. Immune staining of the lung tissue sections for CD4^+^ and CD8^+^ T cells, CD19^+^ B cells and Ly-6G^+^ PMN was performed at weeks 8 (Fig. 2) and 16 (Fig. 3) post-infection, and the following major differences between susceptible and resistant mice were observed.

At the early stage, lungs of genetically resistant I/St mice largely retained an unaltered appearance of normal, highly aerobic breathing tissue. Among the infiltrating cells, only CD4^+^ T lymphocytes (Fig. 2A) and Ly-6G^+^ neutrophils (Fig. 2G) were present in appreciable amounts, whereas CD8^+^ T cells (Fig. 2C) and, particularly, CD19^+^ B cells (Fig. 2E) were scarcely revealed. In genetically susceptible B6 mice, both main populations of T cells (Fig. 2B, D) and neutrophils (Fig. 2H) were present in substantially higher amounts, and started forming cuffs around compact lesions. Visible features of inflammation appeared to be in a good agreement with parameters quantified previously by flow cytometry [15]. Assessment of B-cell infiltration provided the most striking difference between susceptible and resistant mice. In the lungs of B6 mice, B cells were present in high amounts and formed compact, well-boarded foci embedded in areas of pneumonia (Fig. 2F). B-cell foci were totally absent in the lungs of *M. avium-*infected I/St mice (Fig. 2E), whereas such structures were quite common in the lungs of these mice following *M. tuberculosis* infection [10]. We wish to emphasize that the appearance of B cell follicles in the infected mouse lung tissue and formation of structures similar to tertiary lymphoid organs, closely resembling those recently described in humans suffering from advanced TB [22], occur exclusively in animals genetically susceptible to a particular species of mycobacteria. This suggests that this phenotype, both in humans and mice, correlates with an impaired ability to control lung pathology.

As infection progressed, the amounts of lymphoid cells arriving in the lungs of resistant mice were gradually growing (Fig. 3). However, their tissue distribution remained different from that in susceptible animals. Thus, all types of cells displayed more or less diffuse distribution within thickened alveolar septae in I/St mice, with a tendency of CD4^+^ and CD8^+^ T cells cuffing around blood vessels and bronchi (Fig. 3A, C). In contrast, in B6 mice both T cell populations surrounded forming necrotic foci (Fig. 3B, D) that contained huge amounts of dying neutrophils (Fig. 3H), closely resembling necrotic foci previously observed in *M. tuberculosis*-infected I/St mice [10]. B cell follicles in susceptible B6 mice continued occupation of pneumonitic zones distant from necrotic lesions (Fig. 3F).

Previously we compared parameters of lung inflammation following *M. tuberculosis* infection in I/St mice with those in mice of TB-resistant A/Sn strain. These studies demonstrated a marked neutrophil influx on the background of general increase in leukocyte content in the lungs of the former strain [16], [27]. To broaden our knowledge on the dynamics of TB lung inflammation dependent upon genetic susceptibility of the host, we used flow cytomentry to compare accumulation of different leukocyte populations in the lungs of B6 and I/St mice following aerosol infection with 100 *M. tuberculosis* CFU. As shown in Table 1, before challenge mice of the two strains did not differ in the size of any major lung leukocyte population, except a higher CD4:CD8 ratio in I/St mice, which is characteristic for this strain and concerns all anatomical locations, including lymphoid organs (our unpublished observation). Starting week 3 post-infection, significantly more neutrophils accumulated in the lung of TB-susceptible I/St compared to TB-resistant B6 mice. As infection progressed (week 10), lungs of I/St animals accumulated more neutrophils and lymphocytes, indicating progression of inflammatory response, whereas more macrophages arrived in the lungs of B6 mice. Taken together, these results are in agreement with our previous findings, suggesting deleterious rather than protective role of neutrophils in chronic TB infection and important consequences of the difference in mycobacterial distribution between neutrophils and macrophages for immune response and anti-TB defense [27], [29].

**Table 1:** Dynamical changes in the leukocyte content in lungs of B6 and I/St mice following aerosol TB challenge^a^.

Cell population/mouse strain	Weeks post challenge					
	0		3		10	
	B6	I/St	B6	I/St	B6	I/St
Gr1^+^ neutrophils	2.6±0.3	2.6±0.4	3.7±0.4	7.4±1.1*	3.1±0.7	7.1±1.3*
F4/80^+^ macrophages	12.1±1.3	11.9±1.6	13.5±2.0	13.7±2.0	19.0±2.3*	13.0±2.0
All lymphocyte-size gated cells	27.6±4.4	28.5±3.1	31.9±2.7	31.3±3.1	55.9±5.1	66.0±5.7*
CD19^+^ B cells	7.5±2.3	5.4±1.7	8.0±1.6	6.4±2.0	15.3±3.0	13.0±2.4
CD3^+^ T cells	10.4±2.1	11.2±2.7	15.5±2.4	15.5±2.5	30.0±3.0	42.9±5.1*
CD4^+^ T cells	4.2±1.1	6.9±1.0*	8.1±1.7	8.8±1.5	18.6±2.5	21.5±2.8
CD8^+^ T cells	4.7±1.0	3.6±1.0	6.0±1.4	5.7±1.3	10.9±1.6	14.2±2.0

a Results are presented as the proportion (per cent) of a given cell population to the total cellularity of enzymatically digested lung tissue samples. Five to twelve individual samples per group, per time point were analyzed in 2 to 4 separate experiments. Combined means ± SD are displayed.

*Significantly (*P*<0.05, ANOVA) higher than in B6 mice.

**Figure 4:**
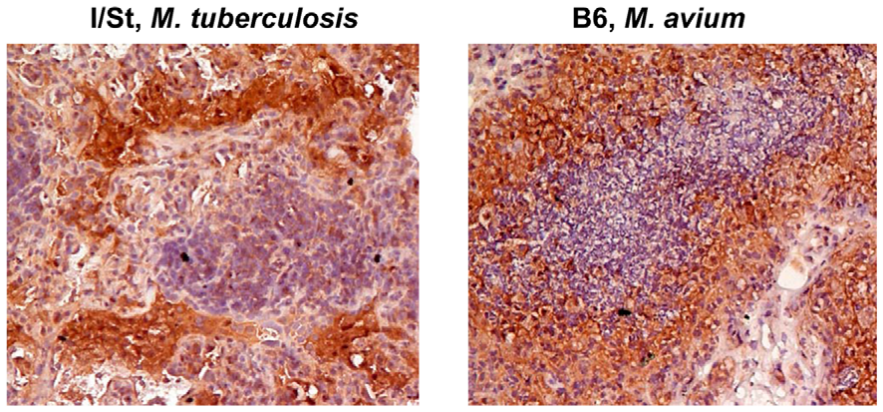
The lung tissue surrounding necrotizing granuloma centers in mice susceptible to *M. tuberculosis* and *M. avium* is markedly hypoxic. I/St mice 6 wk after *M. tuberculosis* challenge (A) and B6 mice 16 wk after *M. avium* challenge (B) were injected with 60 mg/kg body weight of Hypoxyprobe™-1 and sacrificed 3 h later. Lung cryosections were obtained and developed for indirect peroxidase staining to detect hypoxia gradients (×200).

**Figure 5:**
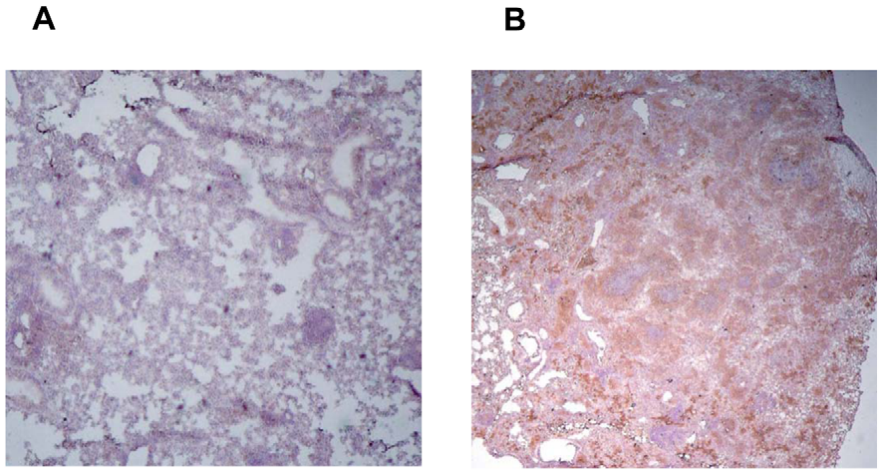
*M. avium* infection does not render active hypoxia in the lungs of resistant mice, but induces numerous necrotizing granuloma surrounded by hypoxic zones in the lungs of susceptible mice. (A)  =  I/St; (B)  =  B6. Hypoxiprobe peroxidase staining (×25).

As mentioned in the Introduction, one of controversial issues concerning the validity of mouse TB models is lung tissue hypoxia which was claimed not to develop in *M. tuberculosis*-infected mice [7], [8], inspiring usage of murine *M. avium* infection for modeling human TB granuloma [12], [13]. To shed light on this problem, we infected groups of I/St and B6 mice with either *M. tuberculosis* or *M. avium* and assessed development of hypoxic zones surrounding disintegrating necrotic foci in the lungs – the type of lesions present exclusively in susceptible but lacking in resistant hosts. As shown in Fig. 4 very similar and quite characteristic hypoxic layers surrounded necrotic granuloma in the lungs of *M. tuberculosis*-infected I/St and *M. avium*-infected B6 mice. Such lesions were totally absent in the lungs of the *M. avium*-infected I/St mice (Fig. 5A), but numerous in the lungs of B6 mice (Fig. 5B). Only few, not regularly shaped, hypoxic zones were noticed in *M. tuberculosis*-infected B6 mice (not shown). Thus, development of circle hypoxic structures in the infected mouse lung appears to be primarily dictated by susceptibility of the host rather than by the nature of mycobacterial pathogen, suggesting that mice genetically susceptible to TB are a reasonable choice to model this human-like phenotype.

### Regulation of neutrophil inflammation

For a long time animal models have failed to demonstrate a clear role of neutrophils in response against mycobacteria, due primarily to conflicting data [23]–[25]. However, more recently we and others using genetic approaches demonstrated deleterious rather than beneficial effects of these early inflammatory cells during mycobacteria-host interactions [26]–[29]. As reported earlier for *M. tuberculosis* [10] and here for *M. avium* (Fig. 3H) infections, massive neutrophil influx into necrotizing inflammatory lung foci is characteristic for mice genetically susceptible to the corresponding mycobacterial pathogen. To look closer on regulation of neutrophil response, we assessed the dynamic expression profiles of the genes encoding major factors involved in neutrophil migration in the inflamed tissues.

**Figure 6:**
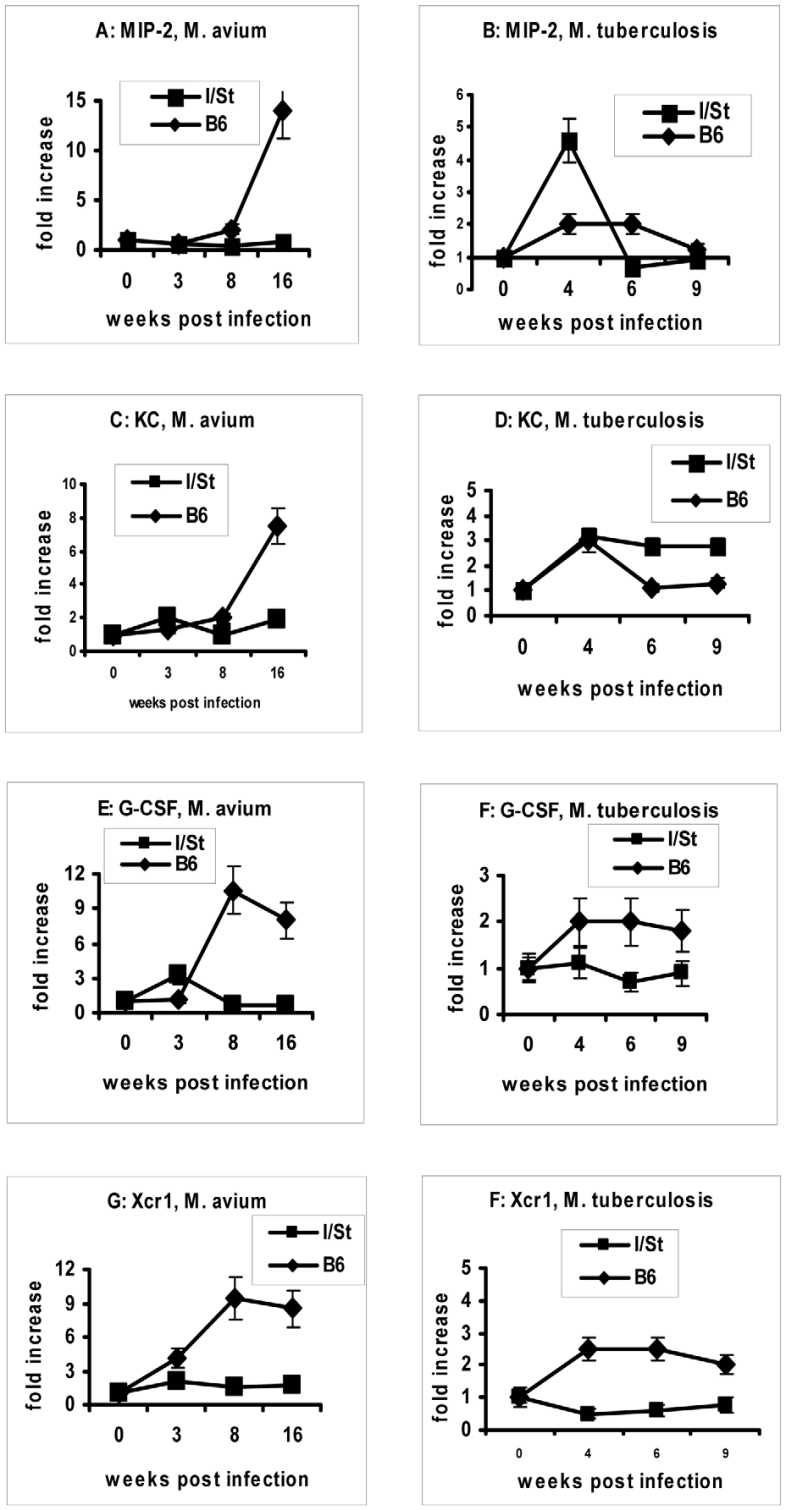
Differences in chemokine gene expression between I/St and B6 mice. At indicated time points samples of RNA were analyzed by the quantitative real-time PCR assay, and gene expression levels in the lung tissue were normalized to those of β-actin (summary of two independent experiments, mixtures of RNA isolated from 3 mice in each, N = 6). Results are expressed as fold increase ± SEM compared to non-infected animals (time  = 0). Factors, infections and mouse strains are marked on each plate.

It is well established that an orchestrated production of two CXC chemokines, KC and MIP-2, as well as the PMN growth factor G-CSF, is required to mobilize neutrophils into inflammatory sites [30], [31]. Progressive *M. avium*-triggered neutrophil inflammation in B6 mice between weeks 8 and 16 of infection was accompanied by a substantial increase in the expression of genes encoding MIP-2 and KC (Fig. 6A, C), on the background of the already elevated G-CSF mRNA levels (Fig. 6E). Expression of the gene encoding Xcr1, a CXC chemokine receptor whose expression on B lymphocytes and neutrophils is up-regulated during inflammation and serves for autocrine amplification [32], [33], displayed similar dynamics (Fig. 6G). In contrast, no to marginal elevations in the expression of all neutrophil chemokine-encoding genes were found in *M. avium*-resistant I/St mice throughout the observation period, which corresponds to a well-controlled inflammatory response.

During *M. tuberculosis* infection the expression profiles were profoundly different. In good agreement with the early peaking neutrophil influx into the lungs of TB-susceptible I/St mice [27], there was a 3- to 5-fold increase in the expression of MIP-2- and KC-encoding genes in their lung tissue at the early phase of infection (Fig. 6B, D). This was, perhaps, sufficient for an early neutrophil accumulation in I/St lungs which is critically dependent upon coordinated up-regulation in the expression of both chemokines [31]. This may also explain a weaker neutrophil response in B6 mice, since there was only marginal up-regulation in the expression of the *mip2* gene in their lungs (Fig. 6B) and a rapid return of the *kc* expression to control levels (Fig. 6D). However, the overall changes in the levels of expression of the genes encoding neutrophil chemokines were less pronounced and showed less clear relations with the interstrain differences in neutrophil inflammation, compared to *M. avium* infection.

We believe that these marked differences in gene expression may be due to substantially different patterns of genetic control of two infections. *M. avium* infection in the mouse strains used in this study is controlled predominantly by the *Nramp1* allelic variation, even in a highly polymorphic, segregating genetic setting [15]. Although this close-to-Mendelian pattern of genetic control is slightly modulated by the *MHC*-linked QTL (N. Logunova, unpublished observations) and, possibly, by the *Hc* locus encoding complement component C5 [34], a major role of a single gene implies relatively straightforward regulatory pathways of host response, including neutrophil inflammation. In contrast, irrespective to the combination of mouse strains, *M. tuberculosis* infection is always controlled by many genetic loci [11], [35], and multiple intercrosses between the resulting regulatory pathways considerably impede simple interpretations. Importantly, researchers never speak about real “resistance” when considering *M. tuberculosis* infection in mice, since, despite huge variations in the speed of disease progression, all mice eventually succumb to infection. In contrast, we recently documented that *M. avium*-infected I/St mice survive more than 14 months, their lung CFU counts drop from ∼10^7^ at week 16 of infection up to ∼10^5^ at month 13, and a normal structure of their lung tissue is restored almost completely. Thus, during *M. avium* infection susceptibility/resistance phenotypes may be delineated even qualitatively, probably, due to a simpler genetic control.

In conclusion, a broad analysis of mycobacterial infections in B6 and I/St mice demonstrates that the level of genetic susceptibility of the host to a given mycobacterial species largely determines characteristics of pathology. Comparative analysis of mycobacterial infections in B6 and I/St mice provides a unique tool based upon “the rule of contraries”, further emphasizing the importance of host genetics in pathogenesis.

## Materials and Methods

### Mice

Mice of inbred strains I/StSnEgYCit (I/St) and C57BL/6JCit (B6) were bred and maintained under conventional, non-SPF conditions at the Animal Facilities of the Central Institute for Tuberculosis (CIT, Moscow, Russia) in accordance with guidelines from the Russian Ministry of Health #755, and under the NIH Office of Laboratory Animal Welfare (OLAW) Assurance #A5502-11. Water and food were provided *ad libitum*. Female mice of 2.5–3.0 mo of age in the beginning of experiments were used. All experimental procedures were approved by the CIT animal care committee (IACUC protocols #2, 6, 8, 11 approved on March 17, 2009).

### Infections

Mice were infected with either virulent *M. avium* strain 724R characterized earlier [36], or standard virulent *M. tuberculosis* strain H37RV (sub-strain Pasteur). Mice were infected via respiratory tract with 1−2×10^3^ viable CFU of *M. avium*, or 1−2×10^2^ CFU of *M. tuberculosis* using an Inhalation Exposure System (Glas-Col, Terre Haute, IN) exactly as described earlier for *M. avium* [15] and *M. tuberculosis* [10]. Isolation and infection of interstitial lung macrophages with *M. avium*, as well as evaluation of their anti-mycobacterial activity, were performed exactly as described earlier for *M. tuberculosis* [18], except that higher multiplicities of infection (MOI) (10∶1–100∶1) were applied due to a lower virulence of *M. avium* compared to *M. tuberculosis*.

### CFU counts and survival time

At indicated time points following infection, lungs from individual mice were homogenized in 2.0 ml of sterile saline, and 10-fold serial dilutions of 0.2 ml samples were plated on Dubos agar (Difco) and incubated at 37°C for 20–22 days before *M. tuberculosis* and *M. avium* CFU were counted. Survival time was monitored daily starting 1 mo post infection.

### Immunohistochemistry

At indicated time points, lung tissue was examined for pathology and infiltration with lymphoid cells. Mice were euthanized by a thiopental overdose. Lung tissue (the middle right lobe) was frozen in the regimen of –60°C to –20°C temperature gradient in the electronic Cryotome® (ThermoShandon, UK), and serial 6–8 µm-thick sections were made across the widest area of the lobe. Lung cryosections were fixed with acetone, blocked with 10% normal mouse serum and stained with peroxidase-conjugated mAbs (all from BD-PharMingen, San Diego, CA) against the surface markers of the major lymphocyte populations, CD4^+^ T cells, CD8^+^ T cells, CD19^+^ B cells, and Ly-6G^+^ neutrophils, to assess the architecture and cellular content of inflammatory foci in the lungs. Slides were developed in DAB solution and counterstained with hematoxylin.

To find out whether lung tissue inflammation in mice is accompanied by development of hypoxic zones, groups of infected mice were injected with 60 mg/kg body weight of Hypoxyprobe™-1 (pimonidazole hydrochloride, Chemicon International, Temecula, CA) and sacrificed 3 h later. Lung cryosections were obtained and developed for indirect peroxidase staining to detect hypoxia gradients [37] according to the manufacturer's instructions, with hematoxylin counterstaining. All slides were examined by an experienced pathologist and photographed using Axioskop 40 microscope and AxioCam MRc 5 camera (Carl Zeiss, Berlin, Germany).

### Gene expression evaluation

Total RNA from the whole lungs of individual mice was isolated using the commercial SV Total RNA Isolation System (Promega, Madison, WI). Reverse transcription of mRNA was carried out as follows: 0.5 µg of oligo(dT)primer in 1 µl volume were added to 11 µl of water containing 2 µg of total RNA and incubated at 70°C for 5 min. Samples were chilled on ice for 3 min, and 8 µl RT mix (4 µl 5x RT-buffer, 2 µl 10 mM dNTPs, 1 µl RNasin Plus RNAase inhibitor and 1 µl M-MLV reverse transcriptase; all components from Promega) were added. The samples were incubated at 42°C for 60 min, followed by 10 min at 70°C to stop reverse transcription. Water was added up to the 200 µl volume.

For detection of the mRNA levels for genes involved in neutrophil response, quantitative real-time RT-PCR (qrt-PCR) with cDNA was performed exactly as described earlier [10]. Specific primers and TaqMan probes were obtained from DNA Synthesis, LLC (Moscow). The PCR reaction was performed in a 25 µl final volume of water containing 2 µl cDNA, 2.5 µl 10X TaqPol buffer (Promega), 1 µl 5 mM dNTPs, 1 µl 10 µM forward and reverse primer mix, 0.5 µl 10 µM TaqMan probe, 0.5 µl Taq DNA polymerase (5 u/µl, Promega). PCR amplifications were performed in triplicates using an identical PCR program for all genes: 5 min at 94°C, followed by 50 cycles alternating 15 seconds at 94°C and 1 min at 60°C. Gene expression levels in the lung tissue of individual mice were normalized to those of β-actin (subsequently re-checked to those of GAPDH). To quantify the results obtained by real-time PCR, the comparative threshold method was used exactly as described [38], with the expression of the results as mean fold increase ± SEM for groups of 4 mice each.
